# A brief sketch across multiscale and comparative neuroanatomical features

**DOI:** 10.3389/fnana.2023.1108363

**Published:** 2023-02-13

**Authors:** Kathleen S. Rockland

**Affiliations:** Department of Anatomy and Neurobiology, Chobanian and Avedisian School of Medicine, Boston University, Boston, MA, United States

**Keywords:** cell types, connections, reorganization, subcellular, asymmetry, homology

## Introduction

In 2018, at the Jerusalem conference which inspired this research topic, I repeated the call for more human neuroanatomy (cf. Crick and Jones, 1993) and talked about the related question of human-specific features. Since then, considerable progress has been made in neuroanatomical techniques (e.g., 3-D visualization of whole brains with or without tissue clearing), although the database for human neuroanatomy is still limited. Increasingly apparent, however, is that human specializations are found at multiple scales, from the subcellular and genetic to the macro- and extra-neural (e.g., Axer and Amunts, 2022). This points to a multifactorial answer to “what makes us human” or, rather, to a slightly different question: how do these factors interact across temporal and spatial scales, and what about that is human-specific? As an indirect approach to this question, this opinion is a brief canvas of what may appear as separate topics, although one can sense the building momentum in the research community toward a unified broad systems-level approach. I have neglected important topics of comparative oscillatory signatures as well as prenatal and early developmental processes and confined discussion largely to features of the cerebral cortex.

### Cortical architecture

A widely shared neocortical feature is the organization of six cellular layers. In humans and also in nonhuman primates (NHP), the cellular supragranular layers are thicker, perhaps in accord with a greater abundance and role of the ipsilateral and contralateral corticocortical connections originating preferentially (but not exclusively) from the upper layers (DeFelipe, 2011; Rockland, 2019).

There are other large-brain species with sophisticated behaviors and social structures; but in striking contrast with humans and NHP, cortical areas in elephants, whales, and other cetaceans are pyramidalized, without a small cell layer 4 (Graic et al., 2022). This is a major difference in terms of input organization. Thalamocortical and other inputs, instead of targeting excitatory local neurons in layer 4 together with dendritic elements in the passage, can be assumed to directly target basal or apical dendrites of pyramidal cells. In addition to the reduction of layer 4, layer 1 is thicker in both elephants and cetaceans.

A pronounced verticality, conspicuous in cellular and myelin preparations, is typical of neocortical areas but with species, area, and individual variations that have not been widely systematized (Molnar and Rockland, 2000; DeFelipe, 2011). For apical dendritic bundles, a relatively identifiable feature, quantitative measures exist for dendritic number and diameter per bundle and for bundle-to-bundle spacing but the area and species census is sporadic (Gabbott, 2003).

### Cell packing density

Neuron density varies across species, where average cell density is least in humans, and progressively more dense in chimpanzees, macaques, rats, and mice (Figure 13 in DeFelipe, 2011). The reduced cell density complements an increase in neuropil space and synaptic density, which would be consistent with more complex neural processing in larger brains. A greater number of neurons has often been posited as correlating with human cognitive abilities; but a less neuron-centric view assigns a prominent role to biochemical variations and interactions of neurotransmitters, neurotrophins, and other substances facilitating communication (cf. Saniotis et al., 2020; Chin et al., 2022).

### Cell types

Broad morphological neuronal subtypes are recognizable across mammalian species; for example, cortical interneurons, cortical pyramidal neurons, and cerebellar Purkinje cells. For the typical cortical pyramidal neurons with apical dendritic tufts in layer 1, species-specific morphometric modifications have been reported in the size of the basal dendritic arbor (Elston, 2003), the length of the apical dendrite (Galloni et al., 2020), the number of dendritic branches, and the number of postsynaptic dendritic spines. These parameters are all significant for electrical properties and microcircuitry processing and are increased in humans and NHP by comparison with rodents (Gilman et al., 2017).

Layer 3 magnocellular pyramidal neurons in the cognitively important human prefrontal cortex have a protracted multi-stage maturational process of the dendritic arbor, with an additional phase of extensive dendritic growth during early childhood (Petanjek et al., 2019). One consequence of the protracted circuitry reorganization with synaptic overproduction and pruning is a great opportunity for gene–environment interactions.

Morphologically distinct cell types, such as Von Economo cells, Betz cells, and Meynert cells, occur in humans but are also reported in NHP, as well as in some non-primate species (Jacobs et al., 2018). Thus, human-specific features may be primarily quantitative in nature and/or at the subcellular level of receptors, ion channels, and input distribution (cf. Hodge et al., 2019). At the transcriptomic level of analysis, still ongoing results so far indicate a high degree of diversity for both interneurons and pyramidal cells (e.g., Galakhova et al., 2022 for a recent discussion). An apparently human-specific GABAeric cell type has been identified in layer 1 (rosehip: Boldog et al., 2018).

In contrast with the relatively subtle morphological differences in neurons, pronounced structural differentiations have been identified for glia; namely, humans have larger and structurally more complex protoplasmic and fibrous astrocytes, and several distinct glia subclasses not so far identified in rodents (Oberheim et al., 2009; Falcone et al., 2021). These variations are functionally significant, as astrocytes are involved in the maintenance of the blood–brain barrier; synapse formation, elimination, and plasticity; calcium flux regulation; and modulation of neural activity. Astrocytes communicate by gap junctions. The increased volume of an astrocytic domain in humans carries an increased synaptic number (humans: 270,000–2 million synapses vs. 20,000–120,000 synapses in rodents); and a larger glial-neuronal unit can be hypothesized as adapted for more complex processing (Oberheim et al., 2009).

### Nano-architecture

The subcellular level of organization has been relatively under-investigated but is an important source of functionally relevant comparative data (Garcia-Etxarri and Yuste, 2021). Ultrastructural analyses have already indicated synaptic structural specializations in humans. Cortical synapses in layer 5 of the human temporal cortex have 2–3 fold larger active zones, a larger pool of synaptic vesicles (releasable, recycling, and resting), and tighter astrocytic ensheathment—all features compatible with greater efficiency (Rollenhagen et al., 2020). Strong and reliable synaptic communication, as compared with the mouse, has been verified by *in vitro* electrophysiology of surgical samples from the human temporal cortex (Hunt et al., 2022).

Cytoskeletal features are being revealed to a new degree of intricacy and diversity, impacting the understanding of neuronal trafficking and axonal transport mechanisms (Leterrier et al., 2017). The axon initial segment, readily identifiable across species, has specific morpho-functional features which are not fixed but changeable in neural activity or pathological conditions (Anton-Fernandez et al., 2022).

The extracellular space is increasingly appreciated as an integral component of brain architecture, forming a complex 3D structure that occupies about 20% of brain volume (Hrabetova et al., 2018). Area and species differences will impact brain fluid dynamics and homeostasis (Rasmussen et al., 2022).

Myelination provides a further rich field for the investigation of species-specific specializations (Fields, 2015). A human-specific hallmark is developmentally prolonged cortical myelination, allowing time for several epochs of reorganization. Data from rodents strongly support the idea of differences in myelin thickness and spacing of nodes of Ranvier in both activity-dependent and -independent conditions (de Faria et al., 2021). Transcriptomic profiles in humans and mice, an important model of human myelin disorders, have been reported as well-correlating but with a divergent expression of distinct genes (Gargareta et al., 2022).

### Asymmetries

Hemispheric asymmetries (e.g., Thiebaut de Schotten and Beckmann, 2022) have been closely associated with human-specific features such as manual dexterity and language; and a left hemisphere (LH) prefrontal hyperscaling is put forth as a neural adaptive shift distinguishing the ape and monkey radiations (Smaers et al., 2011). Other asymmetries, including in humans (Toga and Thompson, 2003), are developmental (the right hemisphere (RH) develops earlier and faster) and neurochemical (higher dopamine levels in the LH and higher adrenaline in the RH). There is a preponderance of cilia-associated genes in the RH but cell adhesion-associated genes in the LH (Lubben et al., 2021). Hemispheric asymmetry has been attributed to multiple mechanisms, both genetic and environmental (Buckner et al., 2009). The right-left asymmetry of frontal and parieto-occipital regions has been referenced to a lateralized gradient of embryological development, specifically in humans (Xiang et al., 2019); and a genetic association has been reported in relation to the typical counterclockwise brain torque (Zhao et al., 2022).

A left-right cerebral torque appears to be human-specific, where the RH has an anterior protrusion but the LH protrudes posteriorly. There is individual variability (40% of subjects exhibit torque), and the torque is reported to be less common in females (Xiang et al., 2019).

Several brainstem nuclei are identified as specific to humans, with species-variable neurochemical differences in homologous nuclei and pronounced left-right asymmetry in humans (Baizer, 2014). These changes in brainstem organization were attributed to the unique human motor and cognitive abilities, as associated with the parallel expansions of cortical and cerebellar structures (Refer to Smaers and Soligo, 2013 on “brain reorganization not relative brain size.”).

### Connectivity

Comparisons between NHP and humans are a rich source of inquiry on human-specific brain organization. Recently, a large proportion of this research is from functional imaging or tractography, not treated here (but refer, for recent review and references, to Thiebaut de Schotten and Forkel, 2022). New data on differential human-specific features of connectivity can be expected from three-dimensional multimodal atlases of large cohorts of human subjects, as compatible with cross-species comparisons (Thiebaut de Schotten et al., 2019).

The clinically important corticospinal (CS) tract serves as a representative example of species differences. The CS tract contains a wide spectrum of fiber diameters, of which a subpopulation of large-diameter fibers (up to 22 vs. 12 μm in macaques) is characteristic of humans (Lemon, 2021). Fast-conducting large fibers are associated with reduced conduction delays which are considered important in movement and skilled grasp transitions (summarized in Lemon, 2021). For a comparable discussion of the corpus callosum, refer to Innocenti et al., 2022.

Another variable is the proportion of crossed fibers: 80–95% in rodents but 75–90% in humans, with noticeable individual variability (Welniarz et al., 2017). Furthermore, a large number of cortical areas contribute to the CS tract in humans, from parts of the frontal, cingulate, and parietal cortices in addition to the primary motor (the latter estimated as 50%; Lemon, 2021). Convergence of these diverse inputs in the spinal cord and their communication by corticocortical connections is likely to subserve other than purely motor functions.

The gray matter terminations of long-distance axons are difficult to investigate in large brains and especially in humans, where standard anatomical techniques cannot be used. There are some data from labeling by antibodies or, increasingly, dense ultrastructural analyses of small tissue volumes in postmortem material or surgical samples. Otherwise, results in experimental animals need to serve as a provisional guide (Rockland, 2020). Likely homologs might be thalamocortical axons in the primary sensory areas, the highly conserved pattern of corticothalamic projections from layers 5 and 6 to non-sensory thalamic nuclei (Rouiller and Welker, 2000), and the divergent collateralization pattern of layer 5 cortico-brainstem neurons (Coude et al., 2018). Organizational differences (e.g., larger arbors and more boutons) can be expected since even homologous structures have modified characteristics. In primates, but not in rodents, the thalamus is densely innervated by dopaminergic fibers (Garcia-Cabezas et al., 2009) and the primate thalamus has both excitatory projection neurons (positive for calbindin and parvalbumin) and inhibitory interneurons, the latter of which are only sparsely present in the rodent thalamus (Arcelli et al., 1997).

In mice, connectivity analysis is approaching routine at the level of whole neuron/whole axon (and Xu et al., 2021 in macaque; McElvain et al., 2021; Gao et al., 2022). A general conclusion from this approach is that individual axons within the same source–target system are highly variable in branching patterns, size of single arbors, and the number of synaptic boutons. This is consistent with smaller sample sizes from previous work in NHP (refer to references in Rockland, 2020).

## Conclusion

In this brief and selective opinion, I have attempted to highlight several examples of human-specific characteristics. There are, of course, many others, such as species differences in the cerebrospinal fluid, lifespan changes in myelination and other parameters, and species characteristics of the brain oscillations and sleep cycles. An important direction is a closer integration of cognitive and systems neuroscience with the cellular, subcellular, and molecular levels of ion channels, receptors, and other components of signal transduction and metabolic pathways (cf. Raichle, 2015 among others).

As a broad systems-level approach will include cultural and environmental influences, a key question is how external factors access and influence neuronal substrates. The influence of external factors has commonly been assayed by changes in dendritic spine density in response to environmental stressors, or aging and pathology (e.g., Jacobs et al., 2022) but can be expected to impact a large number of interacting elements across scales.

## Author contributions

The author confirms being the sole contributor of this work and has approved it for publication.
